# Obesity and Obesity-Related Thyroid Dysfunction: Any Potential Role for the Very Low-Calorie Ketogenic Diet (VLCKD)?

**DOI:** 10.1007/s13668-024-00528-w

**Published:** 2024-03-25

**Authors:** Sebastián Pablo Chapela, Alison Simancas-Racines, Florencia Ceriani, Andrés Luciano Nicolas Martinuzzi, María Paula Russo, Ana Karina Zambrano, Daniel Simancas-Racines, Ludovica Verde, Giovanna Muscogiuri, Christos S. Katsanos, Evelyn Frias-Toral, Luigi Barrea

**Affiliations:** 1 Facultad de Medicina, Departamento de Bioquímica Humana, Universidad de Buenos Aires, Ciudad Autónoma de Buenos Aires, Argentina; 2 Equipo de Soporte Nutricional, Hospital Británico de Buenos Aires, Ciudad Autónoma de Buenos Aires, Argentina; 3 Facultad de, Ciencias Agropecuarias y Recursos Naturales, Carrera de Medicina Veterinaria, Universidad Técnica de Cotopaxi, Latacunga, 050108 Ecuador; 4 Centro de Investigación de Salud Pública y Epidemiología Clínica (CISPEC), Facultad de Veterinaria y Agronomía, Universidad UTE, Santo Domingo, Ecuador; 5 Escuela de Nutrición, Universidad de la República Uruguay, Montevideo, Uruguay; 6 Nutrihome, Buenos Aires, Argentina; 7 Facultad de Medicina, Universidad Nacional del Comahue, Buenos Aires, Argentina; 8 Servicio de Clínica Médica, Hospital Italiano de Buenos Aires, Ciudad Autónoma de Buenos Aires, Argentina; 9 Centro de Investigación Genética y Genómica, Universidad UTE, Facultad de Ciencias de la Salud Eugenio Espejo, Quito, Ecuador; 10 Centro de Investigación de Salud Pública y Epidemiología Clínica (CISPEC), Universidad UTE, Facultad de Ciencias de la Salud Eugenio Espejo, Quito, 170129 Ecuador; 11 Department of Public Health, University of Naples Federico II, Via Sergio Pansini 5, 80131 Naples, Italy; 12 Unità di Endocrinologia, Diabetologia e Andrologia, Dipartimento di Medicina Clinica e Chirurgia, Università degli Studi di Napoli Federico II, Via Sergio Pansini 5, 80131 Naples, Italy; 13 Cattedra Unesco “Educazione Alla Salute E Allo Sviluppo Sostenibile”, University Federico II, 80131 Naples, Italy; 14 School of Life Sciences, Arizona State University, Tempe, AZ 85259 USA; 15 School of Medicine, Universidad Espíritu Santo - Samborondón, 0901952 Samborondón, Ecuador; 16 Dipartimento di Benessere, Nutrizione e Sport, Università Telematica Pegaso, Centro Direzionale Isola F2, Via Porzio, 80143 Naples, Italy

**Keywords:** Very low-calorie ketogenic diet, Hypothyroidism, Obesity, Triiodothyronine, Thyrotropin, Thyroxine

## Abstract

**Purpose of Review:**

This review aims to explore in-depth the different aspects of the association between very low-calorie ketogenic diet (VLCKD), obesity and obesity-related thyroid dysfunction.

**Recent Findings:**

The VLCKD, proposed as a non-pharmacological strategy for the management of certain chronic diseases, is becoming increasingly popular worldwide. Initially used to treat epilepsy, it has been shown to be effective in controlling body weight gain and addressing various pathophysiological conditions. Research has shown that a low-calorie, high-fat diet can affect thyroid hormone levels. Weight loss can also influence thyroid hormone levels. Studies have suggested that long-term use of VLCKD for refractory epilepsy may be related to the development of hypothyroidism, with an effect seen in various populations. In particular, women with obesity following VLCKD tend to have reduced T3 levels.

**Summary:**

We propose further research to unravel the underlying mechanisms linking VLCKD to obesity and obesity-related thyroid dysfunction.

## Introduction

Very low-calorie ketogenic diets (VLCKDs) consists of diets with a higher proportion of calories coming from fat (90%) and a smaller proportion (10%) from carbohydrates and proteins [1]. These diets are becoming increasingly popular dietary interventions not only for weight loss but also for reducing the severity of disease states associated with obesity. This dietary approach is proposed as a non-pharmacological strategy for managing chronic diseases like fatty liver, dyslipidemia, cancer, hypertension, coronary disease, type 2 diabetes, obesity, and its related comorbidities [2••]. Evaluations of its safety and effectiveness have been conducted within the fields of endocrinology, oncology, and neurology [3]. In this context, the adoption of VLCKD is advised by the Italian Society of Endocrinology (SIE) for several obesity-related diseases, including hypertension, dyslipidemia, and type 2 diabetes mellitus [2••]. Additionally, the European Association for the Study of Obesity (EASO) recommends VLCKD as an effective personalized nutritional treatment for patients with obesity [4••].

VLCKDs involve reducing carbohydrate intake while increasing the proportion of calories from fat and protein. Principal differences between VLCKD and other ketogenic diets (KDs) used in clinical practice are shown in Table 1.

**Table 1:** Descriptions and abbreviations for ketogenic diets

Name and Nomenclature	Description
Very-low-calorie ketogenic diet (VLCKD)	Intake of lipids should be less than 30—40 g/day. Protein intake should be about 0.8—1.2 g/day *per* kg of ideal body weight. Carbohydrate intake should be less than 30—50 g/day, while calorie intake should be more than 700—800 kcal/day
Low-calorie ketogenic diet (LCKD)	Intake of lipids should include 30—40 g/day, less than 30—50 g/day of carbohydrates, and over 700—800 kcal/day
Isocaloric ketogenic diet (ICKD)	For a low-carb diet, daily carbohydrate intake should be limited to 30—50 g/day, with calorie intake of at least 700—800 kcal/day and 70—80% of total daily calorie intake

*VLCKD *Very-low-calorie ketogenic diet,* LCKD *Low-calorie ketogenic diet*, ICKD *Isocaloric ketogenic diet

All of these diets have the capacity to exert a beneficial influence on metabolic parameters, such as blood pressure, glycemia, and lipid levels, in addition to weight loss [2••, 5–12]. KD has been employed in the management of epilepsy that is resistant to standard treatments, although with varying degrees of success. While the exact mechanism by which KD improves the health of these patients is not fully understood, it is believed that switching from glucose to ketone body metabolism favorably affects the regulation of certain neurotransmitters, oxidative stress, and ion channels [13–16]. Based on current evidence, the effectiveness of KD extents to various pathologies, including impaired glucose control [17•, 18], obesity [17•, 19, 20], polycystic ovary syndrome [11, 14, 21, 22], cancer [23, 24], and psoriasis [25]. KD has been studied as a tool to preserve muscle mass in the context of weight loss programs [8, 26, 27].

Animal studies have demonstrated that VLCKD inhibits cortisol production, influencing metabolic and endocrine regulation [28]. Additionally, VLCKD can decrease renin while increasing aldosterone levels in patients with obesity, thereby reducing blood pressure [29]. Nevertheless, more studies are needed to provide deeper insights into the underlying biology related to these effects of KD on body weight and overall body physiology [28].

The prevalence of overt hypothyroidism ranges between 0—3% and 3—7% in the general population [30–32]. Hypothyroidism can arise from various factors, including autoimmune disorders, congenital conditions, pregnancy, certain medications, irradiation, thyroidectomy, and iodine deficiency [30–32]. It is noted that a third of the world's population lives in iodine-deficient areas [30–32]. Hypothyroidism can result in significant ramifications, including myxoedema coma and increased risk for heart failure and stroke in younger individuals. While there is certain controversy, it has also been linked to cognitive problems [31]. The evidence suggests that dietary choices, weight loss, and changes in body mass index (BMI) may impact the levels of thyroid hormones. Also, compared to those who have no obesity, the ones with it have a higher prevalence of subclinical hypothyroidism (sHypo) [33]. However, in patients with obesity, the identification and management of sHypo can present challenges. Individuals with euthyroid obesity exhibit higher thyroid-stimulating hormone (TSH), free triiodothyronine (fT3), and fT3/fT4 ratios in comparison to patients without obesity with the same condition [33].

Animal and human studies report an association between diet and T3, thyroxine (T4), and THS levels [34–37]. Specifically, the caloric distribution provided by high fat and low carbohydrate caloric distribution has been shown to affect the thyroid profile [38–40]. In this review, we consolidate evidence of how VLCKD composition and distribution of calories within macronutrients may affect the levels of the hormones T3, total T4 (TT4), and free T4 (fT4). Additionally, we discuss mechanistic links between this diet and thyroid functional status both in health and in different pathological states. Finally, we propose further research to unravel the underlying mechanisms linking VLCKD to obesity and obesity-related thyroid dysfunction.

## VLCKD Nutritional Protocol

The VLCKD protocol involves reducing the daily percentage of carbohydrate intake while increasing those of protein and fat while restricting daily calories to less than 800 kilocalories (kcal) [1, 41]. The diet prioritizes high-biological value proteins, typically from milk, peas, whey, soybeans, and natural or artificial foods. A typical preparation contains approximately 18 g of protein, 4 g of carbohydrates, and 3 g of fat, for a total of 100 to 150 kcal [4••]. Therefore, carbohydrates contribute around 13% of the total energy intake, while fats and proteins comprise about 44% and 43%, respectively [2••]. Fats of vegetable origin high in oleic acid content are prioritized in the diet. As a result of the reduced carbohydrate intake, ketone bodies are produced that are used as fuel by various extrahepatic tissues, such as the heart, skeletal muscles, and the central nervous system [4••].

The three steps of the VLCKD protocol —active, re-education, and maintenance—must be discussed with the patient. Before starting the VLCKD protocol, it is essential to highlight the strict contraindications associated with it. These include moderate renal failure, type 1 diabetes mellitus, latent autoimmune diabetes in adults, sodium/glucose cotransporter 2 inhibitor use, and cell failure in type 2 diabetes. Additionally, chronic to severe kidney disease, liver failure, heart failure (as per the New York Heart Association Classification (NYHA) III-IV), unstable angina caused by respiratory failure, recent myocardial infarction or stroke (within the past 12 months), cardiac arrhythmias, and eating disorders are among some of the conditions where this diet is not recommended [1, 2••]. All three steps mentioned earlier are described below.

## VLCKD Protocol: Step by Step

According to the SIE position statement, the VLCKD protocol has several stages [42–44]. The first 8—12 weeks describe reduced carbohydrate intake to induce nutritional ketosis [2••, 45]. During the initial stage, the diet consists of very few calories (650—800 kcal/day) and is low in carbohydrates (less than 30 g daily from vegetables) and fat (only 20 g *per* day, derived from olive oil). The protein intake of high-biological-value ranges between 1.2 and 1.4 g *per* kilogram of ideal body weight, intending to preserve lean body mass. Protein can be obtained from common foods like eggs, meat, or fish, or can use meal replacements instead [23, 24]. Current scientific evidence supports the use of meal replacements during the first active ketogenic phase. This measure ensures safe, effective, and controlled administration of VLCKD. Tailored meal replacement options offer a more precise calibration of an individual's dietary intake, enabling precise and personalized adjustment of the calorie, macronutrient, and micronutrient content required by the patient [31]. In this respect, the recently published guideline KeNuT, endorsed by the Club of the SIE-Diet Therapies in Endocrinology and Metabolism, provides an overview of the clinical indications, contraindications, mechanisms of action, and management strategies associated with ketogenic nutritional therapy, particularly utilizing meal replacements [41].

Setting a VLCKD protocol that includes meal replacements provides enhanced safety, effectiveness, and adherence in patients with obesity. Because meal replacements are lower in fat and carbohydrates and higher in protein, freeze-dried meal replacements are recommended, resulting in better weight loss and adherence [46]. Moreover, because this is a low-calorie diet (LCD), worldwide standards suggest supporting patients with micronutrients such as vitamins (C, E, and complex B), minerals (magnesium, potassium, calcium, and sodium), and omega-3 fatty acids [46]. Proper hydration is also crucial at this early period, and patients are advised to drink about 2—2.5 L of water *per* day. Vegetables with a low glycemic index must be consumed in order to reach the required daily fiber intake.

Following the initial active ketogenic phase, a LCD is advised, during which time other food categories are gradually resumed. In particular, foods with the lowest glycemic index—like fruits and dairy—are the first to be progressively reintroduced, along with carbohydrates. The LCD diet calls for a daily caloric intake of 1000 – 1200 kcal, along with 60 – 100 g of carbohydrates. After that, a hypocaloric balanced diet is maintained, reintroducing legumes and consuming 1300 – 1400 kcal and 130 – 150 g of carbohydrates [46].

It is essential to maintain a hypocaloric balanced diet that follows the Mediterranean diet with a caloric intake between 1500—2200 kcal in the final maintenance stage [1]. This diet should include low glycemic index cereals. Adopting healthy eating habits is vital for sustaining long-term results effectively [39]. According to research, it is critical for patients with obesity to reduce body weight by at least 15% and maintain that reduced weight in order to decrease their cardiometabolic risk [1, 4••]* per* kg of desirable weight (i.e., weight corresponding to a BMI of 22.5 kg/m^2^). High-fiber foods with slow-absorbing starches are recommended sources of carbohydrates; excessive simple sugar intake (max 10%) should be avoided, and instead, opt for a diet that is abundant in cereals, fruits, vegetables, and legumes. Patients can use this dietary pattern to maintain weight loss while remaining in their nutritional re-education [1, 4••]

The KD can aid in weight loss, insulin sensitivity, and hormonal balance. However, it can cause nutrient deficiencies, as it is characterized by limited food choices, and lacks evidence of association with long-term effects [47]. Conversely, the Mediterranean diet is a nutrient-rich strategy that can be advantageous for heart health and has anti-inflammatory properties. Nonetheless, it might not benefit weight loss and needs to be customized for each individual [48].

The KD and the Mediterranean diet each exhibit distinct hormonal influences that impact the female reproductive system in unique ways [14, 22]. Combining these two nutritional approaches can benefit patients with hormonal disturbances in the reproductive system. It has been demonstrated that losing weight increases ovulation likelihood, enhances assisted reproductive technologies, and lessens pregnancy problems [14]. A recent study by Verde and colleagues demonstrated that strict adherence to the Mediterranean diet prior to starting a VLCKD increases the effectiveness of the VLCKD by helping overweight/obesity-afflicted individuals to lose weight while enhancing their body composition [49]. The authors attributed these outcomes to the existence of bioactive compounds in the Mediterranean diet that may aid in creating an ideal metabolic environment for initiating ketosis [49–52]. It is noteworthy that the last phase (maintenance stage) of the VLCKD diet is a Mediterranean-style dietary approach [4••].

## Hypothyroidism and Obesity

In the medical world, there is continuous discussion on the connection between obesity and hypothyroidism. The issue has become more pertinent as a result of the alarming increase in the rates of global obesity [53]. Patients often perceive obesity as a consequence of thyroid malfunction. It is unclear whether sHypo, as opposed to overt hypothyroidism, is associated with weight gain [54]. Thyroid dysfunction can cause changes in weight, temperature, and energy expenditure, regardless of physical activity [55]. Hypothyroidism is related to a decrease in the body's ability to produce heat, a reduction in metabolic rate, and an increase in BMI and obesity [56].

Some experts suggest that changes in TSH levels may be a consequence of obesity rather than the cause [54]. Recent research has revealed a connection between obesity and thyroid autoimmunity, with the hormone leptin produced by adipose tissue as the primary link [54]. Studies have shown that even small changes in levothyroxine (L-T4) dosage during replacement therapy can cause significant variations in resting energy expenditure in hypothyroid patients. There is insufficient data on the amount of weight gain or loss associated with L-T4 treatment for hypothyroidism [54].

There is an inverse relationship between fT4 and BMI, even when fT4 levels are within the normal range [57]. In individuals who are slightly overweight but still have normal thyroid function, fat accumulation is linked to lower fT4 and higher TSH levels, resulting in an increase in body weight over time [58]. This issue suggests that changes in energy expenditure due to altered thyroid function may be a primary factor leading to an increase in body weight, even with normal feedback regulation [59]. Low fT4 levels and a moderate increase in T3 or fT3 levels, are observed in individuals with obesity [60]. Research shows that fat accumulation is related to increased TSH and fT3 levels, independently of insulin sensitivity and metabolic parameters. Furthermore, in patients with obesity, the fT3 to fT4 ratio is positively associated with BMI and waist circumference [61]. Numerous research findings supported the notion that patients with obesity had higher levels of circulating TSH and fT3 than subjects with normal weights. Interestingly, TSH and fT3 changes were completely reversible following significant weight loss (33% BMI decrease) [62]. These findings underscore the pivotal role of adipocytes in the regulation of TSH and thyroid hormones. These results specifically show that obesity can cause peripheral and central thyroid hormone resistance [33, 62, 63].

On the other hand, the most common abnormality in children with obesity is hyperthyrotropinemia, where the thyroid gland produces too much TSH. Recent studies show that these patients often display Hashimoto's thyroiditis ultrasound pattern, indicating an autoimmune attack on the thyroid gland [64]. Nevertheless, these findings have yet to be associated with the production of thyroid autoantibodies.

It is not currently known what causes changes in thyroid function. However, one theory proposes that increased deiodinase activity results in a higher conversion rate of T4 to T3. This is considered a protective strategy in individuals with obesity to counteract the accumulation of body fat by increasing energy expenditure [64]. In individuals with obesity, a possible reason for decreased tissue responsiveness to thyroid hormones may be due to the reduced signaling of both TSH and thyroid hormones in adipocytes. Therefore, the body may increase the secretion of TSH and fT3 to compensate for this change.

Another potential cause of this phenomenon is high leptin levels, typically found in these patients [62]. The main role of leptin is to communicate to the brain the quantity of energy stored in fat tissues and to decrease appetite and food consumption. Additionally, studies have shown that leptin stimulates the production of pro-thyrotropin-releasing hormone (TRH) in the brain, leading to an increase in TRH and TSH levels [54]. Leptin also increases the activity of deiodinases, supporting thyroid hormone function. Moreover, adipose tissue secretes inflammatory cytokines like tumor necrosis factor-alpha, interleukin-1, and interleukin-6, which hinder the mRNA expression of sodium/iodide symporter and iodide uptake activity [54].

Previous research has mainly focused on examining how changes in BMI can influence TSH and thyroid hormone levels. Most studies have found a positive correlation between BMI values and TSH and fT3 levels, but not with fT4. This finding means that a decrease in BMI is likely to lead to a reduction in TSH and fT3 levels, but it does not affect fT4 [38, 65, 66]. Body weight loss results in a drastic reduction of serum fT3 and TSH [44]. Small lifestyle changes, such as increased physical activity and improved body composition, can lower TSH and fT3 levels without changing BMI [67]. These positive changes reduce inflammation and cytokine secretion, which can negatively impact thyroid function. These findings indicate that elevated TSH levels are primarily due to functional changes in thyroid function rather than autoimmune destruction of thyrocytes [54]. Mild hyperthyrotropinemia may result from obesity, so determining thyroid autoantibody status may help diagnose sHypo in obesity [68]. In a genome-wide association study, 807.000 individuals were analysed searching for an association between BMI, TSH and obesity genomic variations [69]. The authors used a bidirectional 2-sample Mendelian randomization showing that serum TSH did not causally lead to changes in BMI or obesity. Moreover, TSH levels could be significantly elevated by genetically predicted high BMI. Also, BMI could casually increase the fT3 while not significantly affecting the fT4 level [69].

Thyroid morphology can be significantly altered by the ongoing inflammation associated with obesity, as evidenced by considerable changes in thyroid volume and hypoechogenic pattern on ultrasonography [33]. After bariatric surgery, weight loss can reverse these alterations [33]. These structural alterations may be linked to the thyroid gland's increased blood vessel permeability and vasodilation brought on by adipokines [33]. Patients with obesity have a higher risk of thyroid nodules due to increased TSH and adipose tissue synthesis of inflammatory mediators [33].

The effectiveness of L-T4 treatment in inducing weight loss in overt hypothyroidism is limited, and there is no established benefit in sHypo. L-T4 administration to control body weight is only indicated for hypothyroid patients with obesity [54]. When used with prolonged calorie restriction, several thyroid analogs may help individuals with obesity and low T3 lose weight by raising their energy expenditure. Further research is required to determine whether sHypo is causally linked to the development of obesity [68].

### Hypothyroidism, Subclinical Hypothyroidism, Obesity and Metabolic Syndrome

There is conflicting evidence regarding metabolic syndrome and sHypo [33]. A large meta-analysis has shown an increased risk of metabolic syndrome in sHypo [70]. On the other hand, another meta-analysis showed a high degree of heterogeneity among the studies, and it was not conclusive in the relation of both pathologies. However, an association was found between obesity and the incidence of overt and sHypo [71].

Furthermore, it has been noted that individuals with obesity have a greater incidence of sHypo. A cross-sectional study among Chinese adults showed an increased risk of sHypo among females [72]. A recent meta-analysis employing a random-effects model revealed a prevalence of 14,6% in patients with obesity [73]. Numerous studies have examined the relationship between leptin levels and autoimmune thyroid disease in patients with obesity [33]. These studies indicate that leptin levels may be linked to Hashimoto thyroiditis regardless of bio-anthropometric variables and that thyroid peroxidase (TPO) antibodies are more common in this group of patients [74]. Obesity and sHypo were revealed to be causally related by a meta-analysis [75]. Populations with obesity showed a significant correlation with Hashimoto thyroiditis and an elevated incidence of sHypo [75].

Diagnosing sHypo presents challenges due to symptom overlap with obesity and the potential influence of obesity on thyroid morphology and function. Additionally, some drugs used for obesity and metabolic syndrome can affect TSH levels [76]. Furthermore, the optimal reference ranges and treatment thresholds for sHypo in obesity are not well established, and the clinical significance of sHypo is unclear. Therefore, it is suggested to follow the current guidelines for thyroid function testing in patients with obesity, which recommend measuring TSH, fT4, fT3, and TPO antibodies in all individuals with obesity, especially before bariatric surgery [77]. Evaluating thyroglobulin antibodies is optional, as it has a low diagnostic value in the context of obesity [77]. The association between obesity and hypothyroidism is also a matter of debate, and other studies are needed to elucidate the possible causal link and the impact of obesity on the prognosis of hypothyroidism and thyroid cancer. Despite the elevated risk of thyroid nodules in patients with obesity, routine thyroid gland ultrasonography is discouraged due to its perceived lack of cost-effectiveness [33, 77].

**Fig. 1:**
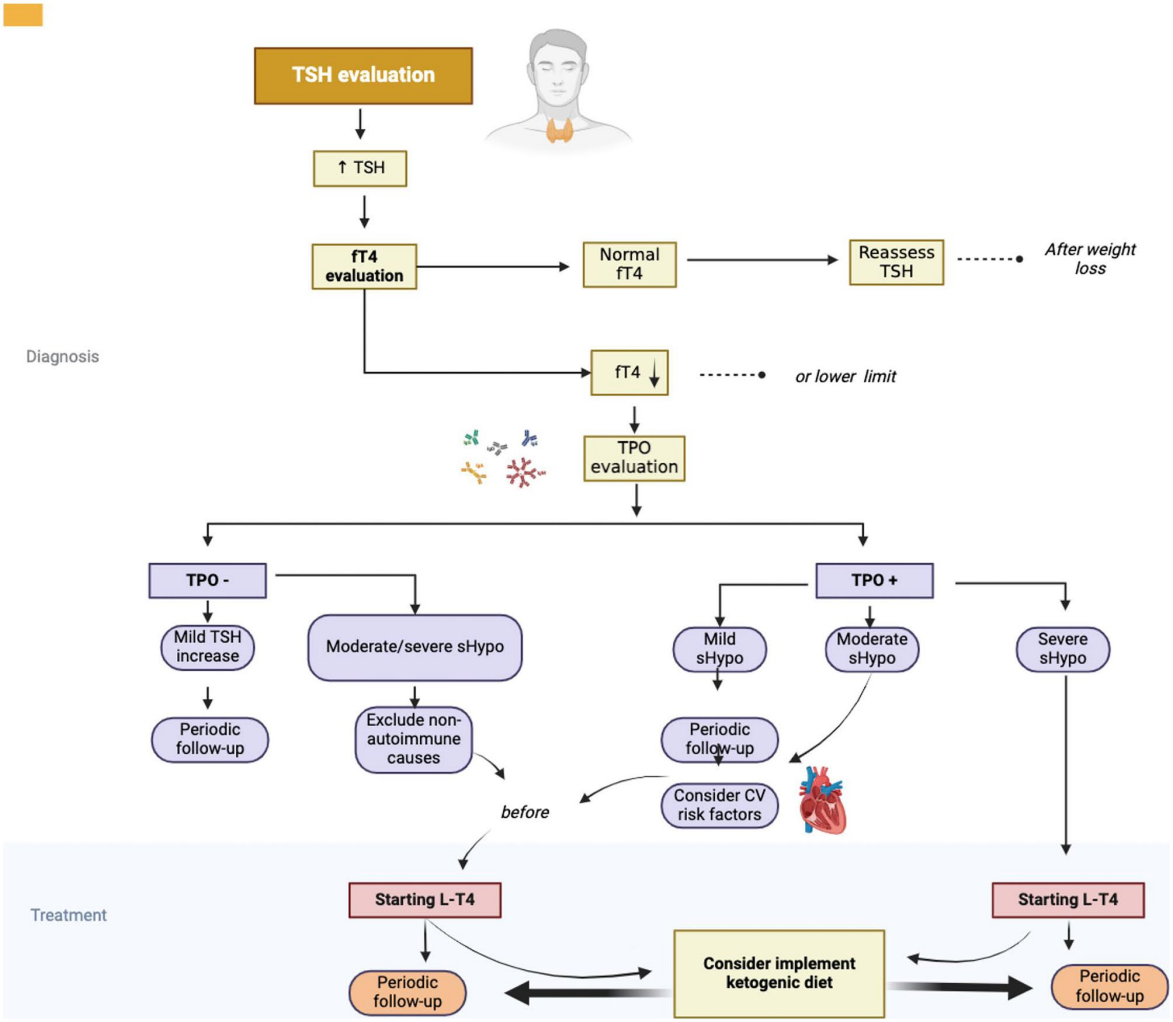
Management and treatment of obesity-related thyroid disorders

The treatment landscape for obesity-related thyroid disorders requires careful consideration of the benefits and risks of L-T4 therapy, as well as the optimal dosage and monitoring of thyroid function [33]. Thyroid hormone preparations, considered for anti-obesity therapy, are now deemed inappropriate due to a lack of supportive evidence and potential adverse effects such as iatrogenic thyrotoxicosis, posing risks to cardiovascular health and metabolic balance [78]. Oral L-T4 therapy is indicated only for patients with obesity with confirmed primary hypothyroidism, especially those with thyroid autoimmunity or other causes of thyroid damage. The dosage of L-T4 should be adapted based on the patient’s BMI, lean body mass, age, sex, and other factors affecting L-T4 absorption and metabolism [79–82]. Moreover, considerations for L-T4 absorption impairment post-bariatric surgery underscore the potential utility of oral liquid formulations to mitigate malabsorption in some instances [83, 84]. In conclusion, personalized and evidence-based approaches are essential in navigating the treatment of obesity-related thyroid disorders, ensuring optimal outcomes while minimizing risks and complications (Fig. 1).

## Hypothyroidism and Diet

**Fig. 2:**
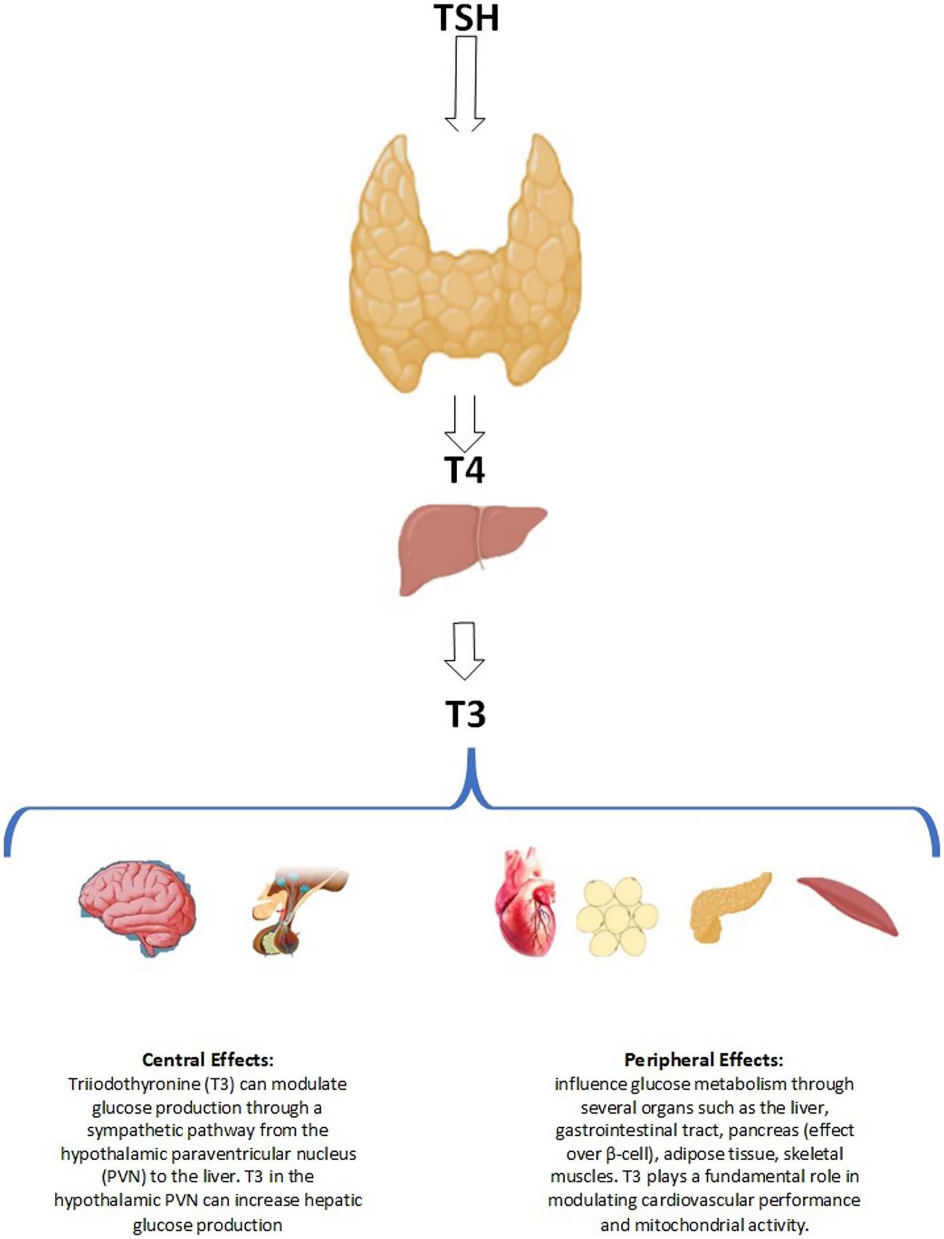
Central and peripheral effects of T3

Thyroid hormones affect glucose metabolism in several organs, including the liver, pancreas (which influences β-cells), the gastrointestinal tract, adipose tissue, skeletal muscles, and the central nervous system [85]. Thyroid hormones increases glucose transporter type 4 (GLUT4) gene expression and glucose uptake in skeletal muscle. T3 enhances the synthesis of glucose in the liver through a sympathetic route originating from the hypothalamus paraventricular nucleus (PVN) to the liver [85] (Fig. 2).

Thyroid function has been studied using animal models to examine the effects of various nutrients. As thyroid hormones can affect lipid metabolism [86], fat intake can affect thyroid function. In one study, Sprague Dawley rats were fed high-fat foods, which increased triglyceride levels and decreased T4 levels. The study also observed changes in the sizes of thyroid gland follicles [40]. Another study found that rats that were fed a high-fat diet rich in saturated and monounsaturated fatty acids for 18 weeks showed hypothyroxinemia, characterized by low TT4 and fT4 levels [39]. The treated rats also had elevated TSH concentration and reduced iodine uptake by the thyroid [38]. Moreover, male Wistar rats fed a high-fat diet (60% fat) had elevated hypothalamic expression of TRH, serum TSH, serum rT3, and iodide uptake in the thyroid gland [38]. These rats also exhibited changes in oxygen consumption and a shift toward fat utilization, as measured by indirect calorimetry. However, this study found no changes in serum T3 and T4 levels [38].

It is crucial to underscore the significant impact of dietary on thyroid hormone levels. As an example, the Mediterranean Diet seems to have a beneficial effect on preventing thyroid diseases, including cancer [86, 87]. Specifically, the synthesis of thyroid hormones depends on iodine. Its deficiency is the most prevalent cause of hypothyroidism in the world [88•]. Iodine intake can be increased by consuming iodized salt, kelp bread, milk, and fish fillets [66]. Alcohol consumption can decrease thyroid volume while increasing TSH levels and reducing fT3 levels [66]. The impact of soy, brassica vegetables, coffee, tea, and junk food on thyroid hormone levels is inconclusive and varies among studies [88•]. A study conducted by Ullrich et al., where 7 healthy volunteers were exposed to a low-carbohydrate diet (35% of total calories) with high protein or fat for 8 days, demonstrated that both diets resulted in a decrease in TSH levels compared to the baseline, with no difference in TSH levels between the two diets [19]. Additionally, both diets led to a decrease in T3 levels from baseline, but the high-fat diet resulted in lower T3 levels [18].

Another cross-sectional study by Brdar et al. involved 4585 healthy individuals from Croatia, who had their plasma-fT3, fT4, and TSH levels determined. The food intake of the subjects was determined with a food frequency questionnaire that evaluated 58 food items [89••]. In the latter study, the levels of fT3 and fT4 were favorably linked to regular consumption of high glycemic index meals and correlated negatively with TSH levels. However, diets high in saturated fats had the opposite effect on fT3 and fT4 levels [89••]. Overall, several studies show that changes in thyroid hormone levels are correlated with caloric intake restriction, weight loss, and, in addition, diet composition, as has been previously discussed [37, 90]. Also, when comparing very low-carbohydrates diets to high- carbohydrates diets, there is a considerable difference in the serum T3 concentrations [91].

The thyroid hormone levels is greatly influenced by the source of the protein that is consumed [92]. It was demonstrated that certain sulfur and aromatic amino acids obtained from the acidic hydrolysis of casein might block TPO’s catalyzed iodide oxidation process *in vitro* [92]. The TPO iodination activity was shown to be impaired by cysteine and methionine or tyrosine and tryptophan; however, their inhibitory potential was reversible at greater iodide concentrations [92]. Additionally, there is proof that the excitatory amino acids, such as aspartate and glutamate, can alter the way the pituitary-thyroid axis secretes hormones [92, 93]

Also, trace elements have shown to have impact on thyroid function. Numerous thyroidal enzymes are selenoproteins, including the glutathione peroxidases that aid in the regulation of oxidative stress in the thyroid cell, and the deiodinases that convert T4 to T3 and rT3 [94]. A higher risk of death and autoimmune thyroid illness are linked to selenium deficiency [94]. Because of this, some have theorized that improving or lowering the likelihood of developing autoimmune thyroid disease may be possible in populations where selenium deficiency exists [94]. Numerous meta-analyses examining the impact of selenium supplementation on autoimmune thyroiditis have revealed decreased TPO antibody titers, whereas neither L-T4 dosage nor TSH levels have changed [94]. TRH and its impact on the pituitary gland, which contributes to the manufacture of TSH, are two of the hormones whose synthesis and function are modulated by zinc [94]. This trace element, also controls the production and concentration of T3 and T4 by moderating the activity of deiodinases [94]. Finally, there are no clear associations between B12 vitamin and autoimmune thyroiditis [94].

## VLCKD in Hypothyroidism

**Fig. 3:**
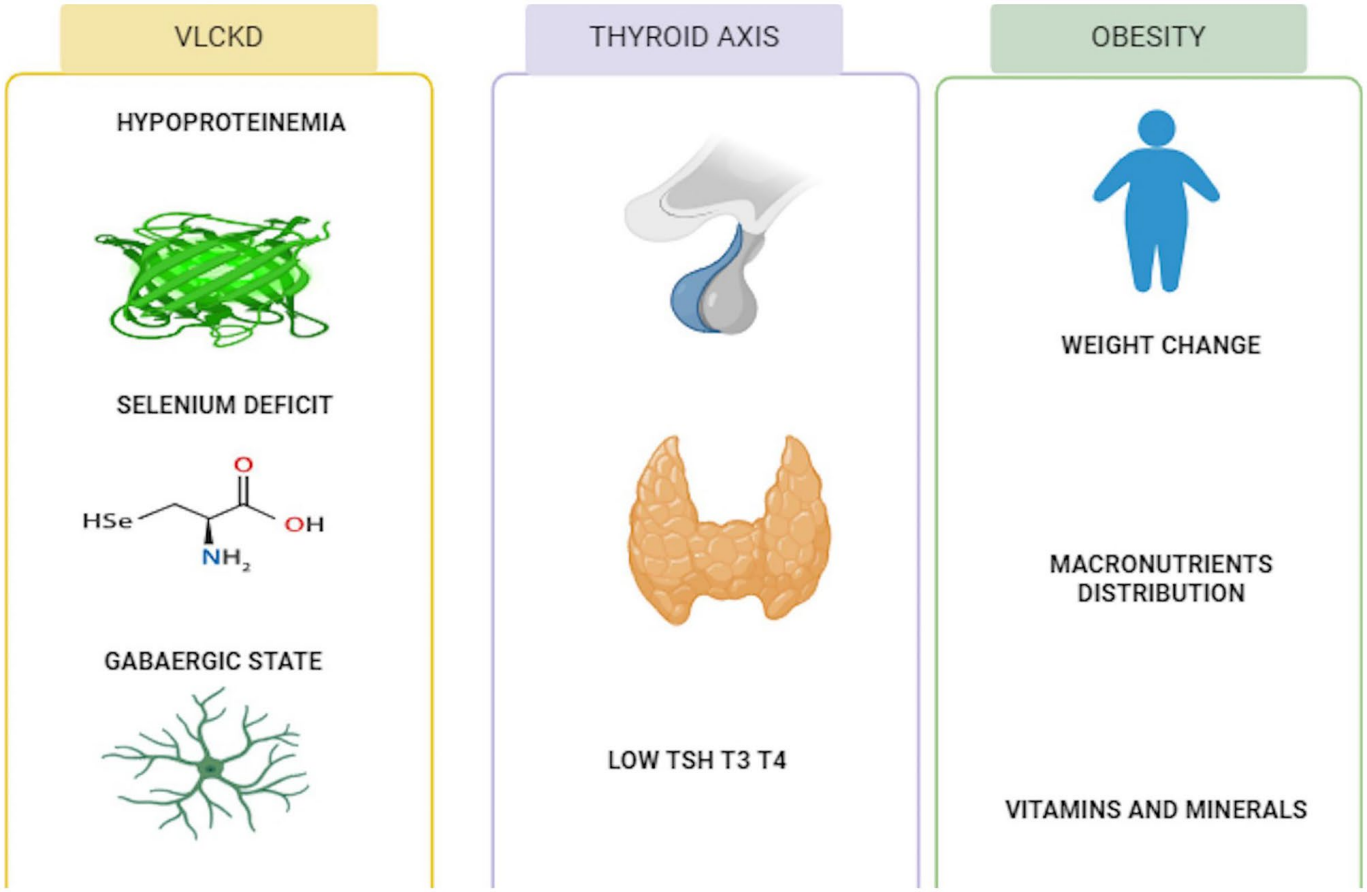
VLCKD in hypothyroidism

The KD diet imitates a state of starvation, causing a shift in metabolism from an insulin-dominant anabolic state to a glucagon-dominant catabolic state. This metabolic shift impacts the thyroid hormones and, as a result, the lean mass, body weight, and dietary carbohydrate intake [91]. This metabolic shift leads to the activation of various metabolic pathways, such as glycogenolysis, gluconeogenesis, lipolysis, and ketogenesis [14]. Research has shown that fasting can decrease the production of T3 and T4 hormones by down-regulating the hypothalamus-pituitary-thyroid axis [95, 96]. Possible mechanisms that may affect thyroid function in patients on VLCKD include selenium deficiency [97], hypoproteinemia [98], and a gabaergic status [99] (as shown in Fig. 3). Generally, diets high in carbohydrates tend to increase the concentration of T3 hormone in the bloodstream compared to low-carbohydrate diets. At the same time, the KD that mimics fasting leads to a significant reduction in T3 hormone levels in the serum. There is often an increase in reverse T3 hormone with this reduction, and it strongly correlates with the presence of ketone bodies [100–102]. Nevertheless, there are scant and contradictory findings about the potential side effects of VLCKD on thyroid function.

A 56-week study using LCD and involving 12 healthy volunteers revealed that, when compared to the baseline, there were notable rises in both total T4 (59.2 ± 11.2 nmol/L *vs* 66.4 ± 12.2 nmol/L) and fT4 index (19.2 ± 3.4 *vs* 21.6 ± 4.6). However, caution should be taken while interpreting the augmented T4 levels, as the researchers did not measure fT3 and T4 [103]. KD can be a suitable long-term therapeutic option for pediatric patients who have intractable drug-resistant epilepsy. However, in such cases, the lower peripheral conversion of T4 to T3 can make children more susceptible to hypothyroidism. Several studies have been conducted to understand potential adverse effects, and according to one of these studies, long-term use of KD can increase the risk of developing hypothyroidism in these patients. Specifically, a study conducted on 120 children with drug-resistant epilepsy examined the effect of VLCKD on thyroid function [95]. Based on their initial characteristics, the patients were assigned into groups with elevated (≥ 5 mIU/L) and normal (< 5 mIU/L) TSH levels [95]. Children with a previous history of thyroid disease or cranial surgery, high TSH levels before VLCKD initiation, and a history of thyroid illness in the family were excluded [95]. Logistic regression analysis revealed that a higher baseline TSH level and being female were independent risk factors for developing hypothyroidism during VLCKD therapy in children with refractory epilepsy. During the first year of treatment, hypothyroidism was diagnosed in 20 out of 120 patients who needed L-T4 supplementation [95].

A study conducted in Turkey in 2021 involved 66 children with drug-resistant epilepsy who were treated with a VLCKD for a minimum of 12 months. The results of the study suggested that there were no noteworthy alterations in the medium serum fT4 or TSH concentrations during the study period [104]. The same study also included four children with pre-existing thyroid function abnormalities who received thyroid replacement therapy from the beginning due to sHypo. The study found that these children were safely treated with VLCKD [104]. Another study on 28 children with VLCKD refractory epilepsy evaluated the prevalence of hypothyroidism and associated changes in the thyroid hormone levels [95]. No discernible longitudinal variation was seen in the mean fT4 and TSH levels between KD initiation and the last follow-up (12 months) [99]. However, patients with a younger seizure onset, earlier initiation of VLCKD, and higher serum triglyceride cholesterol levels showed a notable reduction in fT4 levels and an elevation in TSH levels [99]. A limitation of this study is that it did not include a control group of children with drug-resistant epilepsy who did not undergo a VLCKD. Nevertheless, with a control group, children would not have taken anticonvulsant drugs, which may have influenced the study's results. Yet, it can be concluded that VLCKD has a very minimal or no effect on thyroid function in children with refractory epilepsy. Nonetheless, it is recommended to closely monitor the serum levels of fT4/TSH, particularly in children who start KD at an earlier time, have an earlier onset of seizures, have higher lipid profiles, have higher baseline TSH, and are female. In most cases, replacement therapy is unnecessary, but laboratory hormone findings can be normalized when it is initiated. In a real-world multi-center study, a VLCKD program was implemented to investigate its effects on women with overweight or obesity [7]. Individuals with pre-existing thyroid abnormalities were not included in the study, and no changes in thyroid function were detected throughout the 16-week program [7].

Regarding the effect of VLCKD on the thyroid function of healthy individuals, in 2022, Iacovides et al. addressed the effects of two types of diets on thyroid function and body mass in eleven healthy participants [17•]. To assess the impact of diet on body mass and thyroid function, they conducted a randomized, crossover study with two isocaloric diets: a low-carb diet and a VLCKD [17•]. In response to the two diets, plasma TSH and T4 levels remained constant. However, plasma T3 concentrations decreased more on VLCKD than on the low carb diet (*p* = 0.003), and it was associated with more significant body mass loss [17•]. The study suggests that nutritional ketosis may shift in the T3:T4 circulating ratio, resulting in an increase in inactive T4 and a decrease in active T3 without a change in TSH. The underlying mechanisms of these hormonal changes remain to be understood [17•].

In 2019, the SIE conducted a systematic review and created a consensus regarding using VLCKD to manage metabolic disorders [2••, 3]. The review found no evidence for or against using VLCKD in hypothyroidism but suggested that thyroid function should be monitored throughout the VLCKD regimen. If a patient has uncontrolled hypothyroidism, it is crucial to consider the potential risks of using VLCKD, including dyslipidemia, osteoporosis, depression, gastrointestinal effects, and cardiomyopathy, as these are adverse effects that have been reported [2••].

Several studies have reported detailed effects of VLCKD on the metabolic and endocrine systems (Table 2). In summary, studies conducted on patients with refractory epilepsy have shown that the KD does not affect hormone levels and is safe for patients with hypothyroidism. However, some studies have found that 16.6% of patients with refractory epilepsy and VLCKD have sHypo. Before starting a KD, individuals with an elevated TSH have an increased risk of developing hypothyroidism. Furthermore, research shows a correlation between the KD, weight loss, and changes in thyroid hormone levels. Additional investigation is required to understand the effects of how KD affects thyroid function. A randomized trial is currently being carried out to study the effects of KD and a high carbohydrate diet on sleep and thyroid function. This research will improve our comprehension of how thyroid hormone levels change with the KD [105].

**Table 2:** Main results on thyroid function of the ketogenic diet

Author	Type of Study	Clinical setting and number of participants	Age (Mean ± SD) Sex (male/female)	Composition of ketogenic diet	Duration	Result on thyroid function
Iacovides et al. [17•]	Randomized crossover-controlled study	Healthy and normal-weight (BMI: 24.0 ± 2.0 kg/m^2^) N = 11	Age: 30 ± 9 years Male = 1 Female = 10	Isocaloric dietary interventions: HCLF diet: 55% carbohydrate, 20% fat, 25% protein) KD: 15% carbohydrate, 60% fat, 25% protein	A minimum of three weeks on each diet, with a one-week washout (habitual diet) between the diets	Compared to baseline levels, the change in plasma T3 concentration was significantly different between the two diets (*p* = 0.003). Plasma T3 concentration was significantly lower following the KD diet (4.1 (3.8—4.4) pmol/L, *p* < 0.0001) but not different following the HCLF diet (4.8 (4.5—5.2) pmol/L, *p* = 0.171). There was a significant increase in T4 concentration from pre-diet levels following the KD diet (19.3 (17.8—20.9) pmol/L, *p* < 0.0001), but not following the HCLF diet (17.3 (15.7—18.8) pmol.L, *p* = 0.28). The magnitude of change in plasma T4 concentration was not different between the two diets (*p* = 0.4). There was no effect of diet on plasma TSH concentration (*p* = 0.27). There was a significantly greater T3:T4 ratio following the HCLF diet (0.41 (0.27—0.55), *p* < 0.0001) compared to pre-diet levels but not following the KD diet (0.25 (0.12—0.39),* p* = 0.80)
Tragni et al. [7]	Multi-center, prospective, uncontrolled trial in a real-life setting	Overweight or obesity (BMI: 30.9 ± 2.7 kg/m^2^) N = 44	Age 49.5 ± 7.2 years Only female	VLCKD multi-step dietary model Phase 1: 700 kcal (50 g/day of CHO); 4 weeks Phase 2: 820 kcal (50 g/day of CHO); 4 weeks Phase 2: 1100 kcal (50 g/day of CHO); 4 weeks Phase 2: 1250 kcal (50 g/day of CHO); 4 weeks	Total intervention duration of 24 weeks	TSH: Baseline: 2.40 ± 0.77 mUI/L, Post-VLCKD: 2.31 ± 0.86 mUI/L; Absolute Change (% Change): − 0.09 (− 3.8), *p* = 0.629 Thyroid function markers with no change
Yılmaz et al. [106•]	Retrospectively reviewed	Children with drug-resistant epilepsy N = 66	Aged 3—193 months (median, 52 months) Male = 35 Female = 31	KD was started at a 3:1 ratio with a non-fasting gradual initiation protocol and the ratio was then adjusted between 2:1 and 4:1 as needed to maintain ideal ketone levels for seizure control and minimize adverse effects	KD for at least 12 months	No significant changes in fT4 and TSH concentrations, nor in the number of patients with low fT4 and high TSH concentrations. However, four patients who received L-T4 replacement therapy had increase in serum fT4 levels: and an insignificant decrease in TSH concentrations It appears that ketogenic diet therapy does not impair thyroid functions in children with drug-resistant epilepsy. In addition, KD can be safely used even in children with pre-existing subclinical hypothyroidism along with L-thyroxine replacement
Khodabakhshi et al. [112]	Randomized controlled open-label clinical trial	Locally advanced or metastatic breast cancer and without a history of renal disease or diabetes N = 80	Age: 44.8 ± 8.4 year (KD group) 45.2 ± 15.0 years (control group) Only female	Eucaloric dietary interventions: KD: 6% CHO, 19% protein, 20% MCT oil, and 55% fat Control group: standard diet consisting of 55% CHO, 15% protein, and 30% fat	12 weeks	Thyroid markers with no change
Gomez-Arbelaez et al. [113]	Open, uncontrolled, nutritional intervention clinical trial	Patients with obesity N = 20	Age: 47.2 ± 10.2 year Male = 8 Female = 12	VLCKD multi-step dietary model: The first three steps 600–800 kcal/day, < 50 g daily from vegetables and lipids (only 10 g of olive oil per day. The amount of high-biological-value proteins ranged between 0.8 and 1.2 g per each kg of ideal body weight In steps 4 and 5: 800–1500 kcal/day Step 6: 1500 and 2000 kcal/day and the target was to maintain the weight lost and promote healthy life styles	Patients followed the different steps of the method until they reach the target weight or up to a maximum of 4 months of follow-up	TSH and fT4 did not significantly change, free T3 had a significant although expected decrease
Lee et al. [99]	Retrospective longitudinal cohort study	Children with medically intractable epilepsy N = 28 children	Age at start of KD 3.2 ± 2.4 (0.5–9.9) Boys = 17 Girls = 11	Ratio of KD 4:1/3:1/2:1/1:1	Mean duration of KD was 1.9 ± 1.5 years	There was no significant longitudinal change in the mean fT4 (0.99 ± 0.25 *vs.* 0.94 ± 0.71 ng/dL, *p* = 0.28) and TSH (2.94 ± 1.32 *vs*. 3.18 ± 1.21 μIU/mL, *p* = 0.44) levels from the start of the KD to last follow-up The patients with a younger age of seizure onset the earlier initiation of KD had a significant decrease in fT4 levels and increase in TSH levels during the KD Sex, duration of the seizure or KD therapy, seizure types, seizure frequency, seizure outcomes, brain lesion, ratio of KD, and being overweight did not affect the longitudinal change of fT4 and TSH levels during KD However, it is advisable to carefully monitor the serum levels of fT4/TSH in children on KDs, particularly in those who had an earlier onset of seizures, commenced KD treatment earlier, or have higher levels of lipid profiles
Kose et al. [95]	Single-center design clinical trial	Children receiving KD for at least one year due to drug-resistant epilepsy N = 120 patients	Age: 7.3 ± 4.3 years Males = 63 Female = 57	All children were started on a 3:1 or 4:1 KD ratio [fat/(protein plus carbohydrate)]. A Mediterranean-style KD was prepared with extra virgin olive oil as the principal fat source and common, locally available food as described before	The mean duration of KD was 14.5 ± 3.9 months [12 (12–18 months)]. Maximum duration of follow-up on KD was 24 months (9 patients)	Hypothyroidism was diagnosed and L-T4 medication was initiated for eight, seven and five patients (20 patients in total, 16.7%) at 1, 3, and 6 months of KD therapy, respectively Baseline TSH elevation [OR: 26.91, 95% CI: 6.48–111.76, p < 0.001] and female gender (OR: 3.69, 95% CI 1.05–12.97, p = 0.042) were independent risk factors for development of hypothyroidism during KD treatment in epileptic children KD causes thyroid malfunction and L-thyroxine treatment may be required. TSH elevation and female gender were independent risk factors for developing hypothyroidism in epileptic children during KD treatment

*SD *standard deviation, BMI body mass index,* HCLF *High carbohydrates and low-fat diet,* KD *Ketogenic Diet,* T4, *thyroxine,* T3 *triiodothyronine,* VLCKD Very *low-calorie ketogenic diet,* CHO *carbohydrate,* fT4 *free T4*, L-T4 *levothyroxine*, MCT *Medium Chain Triglycerides,* OR *odds ratio,* CI *confidence interval

## Discussion

This review explores how dietary components relevant to KD can influence thyroid hormone levels. High glycemic index diets have a positive correlation with fT3 and fT4 levels in healthy subjects, while high-fat diets have a negative correlation with the same hormones. Weight loss and changes in BMI can also affect thyroid hormone levels, with weight and BMI correlating positively with T3 levels [66, 88•, 89••, 39, 40].

After reviewing the scarce available evidence, the following conclusion can be drawn. A KD can affect thyroid hormone levels in patients with refractory epilepsy, with up to 16.6% of sHypo [95], although this is not a consistent finding [106•]. It is worth mentioning that many of the usual drugs prescribed to these patients may also affect thyroid hormone levels. One theory is that altered thyroid hormone levels could be a form of euthyroid sick syndrome. This syndrome is characterized by low serum T3 and normal or low levels of TSH, increased serum reverse T3, and with or without decreased T4 [107]. The syndrome is described as an adaptive mechanism to stress that seeks to reduce energy requirements [107].

Levels of T3 appear to be negatively regulated by inflammatory stress like critical illness or chronic inflammatory states like obesity [107]. A KD or a VLCKD could switch off metabolic needs in the periphery, leading to a lesser transformation of T4 to T3. However, in some cases, a VLCKD can increase inflammation [1, 32]. Nonetheless, VLCKD has been shown to decrease high-sensitivity C-reactive protein levels and associated oxidative stress [108].

Several theories have been described regarding the relationship between VLCKD and thyroid hormone levels. Some related changes in obesity, like the leptin hormone, secreted by adipose tissue, can affect TRH release [109]. Evidence shows that this pathway can be mediated by melanocortin [99, 110]. Other theories associated with disturbances in thyroid activity are mechanisms related to selenium deficiency [49], hipoproteinemia 3 [111], and a gabaergic status to disturbances in thyroid activity [95].

Despite the current evidence discussed in this review, there is a need for further research aiming to enhance our understanding of the relationship between VLCKD and thyroid profile. Scientific societies, such as the SIE, recommend using this diet as it is safe even in patients with hypothyroidism but with adequate monitoring [2••, 5]. However, a caveat related to this recommendation is that in patients with uncontrolled hypothyroidism, it can worsen the patient's clinical situation.

## Conclusion

Based on what is discussed in this review, the intake of carbohydrates and fat can affect thyroid hormone levels, in addition to the presence of concurrent weight loss. Moreover, concurrent changes associated with selenium levels and gabaergic status can also affect the thyroid profile. Studies with patients on a VLCKD show that it can chronically affect these thyroid hormone levels, but these studies are limited to specific populations with unique characteristics. Overall, the evidence currently supports using VLCKD as they can mediate favourable outcomes. Moreover, VLCKD diets are safe, but focused research is necessary to assess the prolonged impact of this diet across diverse populations.

## Data Availability

No datasets were generated or analysed during the current study.

## Abbreviations List

VLCKDs: Very low-calorie ketogenic diets
SIE: Italian Society of Endocrinology
EASO: European Association for the Study of Obesity
KDs: Ketogenic diets
BMI: Body mass index
sHypo: Subclinical hypothyroidism
TSH: Thyroid-stimulating hormone
fT3: Free triiodothyronine
T4: Thyroxine
TT4: Total T4
fT4: Free T4
kcal: Kilocalories
LCD: Low-calorie diet
L-T4: Levothyroxine
TRH: Thyrotropin-releasing hormone
TPO: Thyroid peroxidase
GLUT4: Glucose transporter type 4

## References

[1] Muscogiuri G, Barrea L, Laudisio D, Pugliese G, Salzano C, Savastano S, Colao A (2019). The Management of Very Low-Calorie Ketogenic Diet in Obesity Outpatient Clinic: A Practical Guide. J Transl Med.

[2] Caprio M, Infante M, Moriconi E, Armani A, Fabbri A, Mantovani G, Mariani S, Lubrano C, Poggiogalle E, Migliaccio S (2019). Very-Low-Calorie Ketogenic Diet (VLCKD) in the Management of Metabolic Diseases: Systematic Review and Consensus Statement from the Italian Society of Endocrinology (SIE). J Endocrinol Invest.

[3] Trimboli P, Castellana M, Bellido D, Casanueva FF (2020). Confusion in the Nomenclature of Ketogenic Diets Blurs Evidence. Rev Endocr Metab Disord.

[4] Muscogiuri G, El Ghoch M, Colao A, Hassapidou M, Yumuk V, Busetto L (2021). European Guidelines for Obesity Management in Adults with a Very Low-Calorie Ketogenic Diet: A Systematic Review and Meta-Analysis. Obes Facts.

[5] Moriconi E, Camajani E, Fabbri A, Lenzi A, Caprio M (2021). Very-Low-Calorie Ketogenic Diet as a Safe and Valuable Tool for Long-Term Glycemic Management in Patients with Obesity and Type 2 Diabetes. Nutrients.

[6] Rinaldi R, De Nucci S, Castellana F, Di Chito M, Giannuzzi V, Shahini E, Zupo R, Lampignano L, Piazzolla G, Triggiani V (2023). The Effects of Eight Weeks’ Very Low-Calorie Ketogenic Diet (VLCKD) on Liver Health in Subjects Affected by Overweight and Obesity. Nutrients.

[7] Tragni E, Vigna L, Ruscica M, Macchi C, Casula M, Santelia A, Catapano AL, Magni P (1804). Reduction of Cardio-Metabolic Risk and Body Weight through a Multiphasic Very-Low Calorie Ketogenic Diet Program in Women with Overweight/Obesity: A Study in a Real-World Setting. Nutrients.

[8] Paoli A, Speranza E, Vargas Molina S, Caprio M, Camajani E, Feraco A, Proietti S, Basciani S, Barrea L, Armani A (2022). Very Low Calorie Ketogenic Diet Combined with Physical Interval Training for Preserving Muscle Mass during Weight Loss in Sarcopenic Obesity: A Pilot Study. TYPE Orig. Res. Publ..

[9] Barrea L, Cacciapuoti S, Megna M, Verde L, Marasca C, Vono R, Camajani E, Colao A, Savastano S, Fabbrocini G, et al. The effect of the ketogenic diet on acne: Could it be a therapeutic tool? Crit Rev Food Sci Nutr. 2023;1–20. 10.1080/10408398.2023.2176813. 10.1080/10408398.2023.2176813 36779329

[10] Barrea L, Verde L, Camajani E, Cernea S, Frias-Toral E, Lamabadusuriya D, Ceriani F, Savastano S, Colao A, Muscogiuri G (2023). Correction: Ketogenic Diet as Medical Prescription in Women with Polycystic Ovary Syndrome (PCOS). Curr Nutr Rep.

[11] Barrea L, Verde L, Camajani E, Cernea S, Frias-Toral E, Lamabadusuriya D, Ceriani F, Savastano S, Colao A, Muscogiuri G (2023). Ketogenic Diet as Medical Prescription in Women with Polycystic Ovary Syndrome (PCOS). Curr Nutr Rep.

[12] Di Rosa C, Lattanzi G, Taylor SF, Manfrini S, Khazrai YM (2020). Very Low Calorie Ketogenic Diets in Overweight and Obesity Treatment: Effects on Anthropometric Parameters, Body Composition, Satiety, Lipid Profile and Microbiota. Obes Res Clin Pract.

[13] Martin-McGill K, Bresnahan R, Levy R, Cooper P (2020). Ketogenic Diets for Drug-Resistant Epilepsy ( Review ). Cochrane Database Syst Rev Ketogenic.

[14] Kuchkuntla AR, Shah M, Velapati S, Gershuni VM, Rajjo T, Nanda S, Hurt RT, Mundi MS (2019). Ketogenic Diet: An Endocrinologist Perspective. Curr Nutr Rep.

[15] Lima PA, de Brito Sampaio LP, Damasceno NRT (2015). Ketogenic Diet in Epileptic Children: Impact on Lipoproteins and Oxidative Stress. Nutr Neurosci.

[16] Cervenka MC, Patton K, Eloyan A, Henry B, Kossoff EH (2014). The Impact of the Lipid Profiles in Adults with Epilepsy. Nutr. Neurosci..

[17] Iacovides S, Maloney SK, Bhana S, Angamia Z, Meiring RM (2022). Could the Ketogenic Diet Induce a Shift in Thyroid Function and Support a Metabolic Advantage in Healthy Participants? A Pilot Randomized-Controlled-Crossover Trial. PLoS ONE.

[18] Ullrich IH, Peters PJ, Albrink MJ (1985). Effect of Low-Carbohydrate Diets High in Either Fat or Protein on Thyroid Function, Plasma Insulin, Glucose, and Triglycerides in Healthy Young Adults. J Am Coll Nutr.

[19] Gjuladin-hellon T, Davies IG, Penson P, Baghbadorani RA, Amiri Baghbadorani R (2018). Effects of Carbohydrate-Restricted Diets on Low-Density Lipoprotein Cholesterol Levels in Overweight and Obese Adults : A Systematic Review and Meta-Analysis. Nutr Rev.

[20] Schwingshackl L, Hoffmann G (2013). Comparison of Effects of Long-Term Low-Fat vs High-Fat Diets on Blood Lipid Levels in Overweight or Obese Patients: A Systematic Review and Meta-Analysis. J Acad Nutr Diet.

[21] Masi D, Spoltore ME, Rossetti R, Watanabe M, Tozzi R, Caputi A, Risi R, Balena A, Gandini O, Mariani S (2022). The Influence of Ketone Bodies on Circadian Processes Regarding Appetite, Sleep and Hormone Release : A Systematic Review of the Literature. Nutrients.

[22] Pandurevic S, Mancini I, Mitselman D, Magagnoli M, Teglia R, Fazzeri R, Dionese P, Cecchetti C, Caprio M, Moretti C (2023). Efficacy of Very Low-Calorie Ketogenic Diet with the Pronokal® Method in Obese Women with Polycystic Ovary Syndrome: A 16-Week Randomized Controlled Trial. Endocr Connect.

[23] Plotti F, Terranova C, Luvero D, Bartolone M, Messina G, Feole L, Cianci S, Scaletta G, Marchetti C, Di Donato V (2020). Diet and Chemotherapy: The Effects of Fasting and Ketogenic Diet on Cancer Treatment. Chemotherapy.

[24] Aggarwal A, Yuan Z, Barletta JA, Lorch JH, Nehs MA (2020). Ketogenic Diet Combined with Antioxidant N-Acetylcysteine Inhibits Tumor Growth in a Mouse Model of Anaplastic Thyroid Cancer. Surgery.

[25] Barrea L, Caprio M, Camajani E, Verde L, Elce A, Frias-Toral E, Ceriani F, Cucalón G, Garcia-Velasquez E, El Ghoch M, et al. Clinical and nutritional management of very-low-calorie ketogenic diet (VLCKD) in patients with psoriasis and obesity: a practical guide for the nutritionist. Crit Rev Food Sci Nutr. 2022;1–17. 10.1080/10408398.2022.2083070. 10.1080/10408398.2022.2083070 35653127

[26] Cipryan L, Litschmannova M, Maffetone PB, Plews DJ, Dostal T, Hofmann P, Laursen PB (2022). Very Low-Carbohydrate High-Fat Diet Improves Risk Markers for Cardiometabolic Health More Than Exercise in Men and Women With Overfat Constitution: Secondary Analysis of a Randomized Controlled Clinical Trial. Front Nutr.

[27] Barrea L, de Alteriis G, Muscogiuri G, Vetrani C, Verde L, Camajani E, Aprano S, Colao A, Savastano S (2022). Impact of a Very Low-Calorie Ketogenic Diet (VLCKD) on Changes in Handgrip Strength in Women with Obesity. Nutrients.

[28] Barrea L, Verde L, Camajani E, Šojat AS, Marina L, Savastano S, Colao A, Caprio M, Muscogiuri G (2023). Effects of Very Low-Calorie Ketogenic Diet on Hypothalamic-Pituitary-Adrenal Axis and Renin-Angiotensin-Aldosterone System. J Endocrinol Invest.

[29] Barrea L, Verde L, Santangeli P, Lucà S, Docimo A, Savastano S, Colao A, Muscogiuri G (2023). Very Low-Calorie Ketogenic Diet (VLCKD): An Antihypertensive Nutritional Approach. J Transl Med.

[30] Chaker L, Bianco AC, Jonklaas J, Peeters RP (2017). Hypothyroidism. Lancet (London, England).

[31] Chaker L, Razvi S, Bensenor IM, Azizi F, Pearce EN, Peeters RP (2022). Hypothyroidism Nat Rev Dis Prim.

[32] Taylor PN, Albrecht D, Scholz A, Gutierrez-Buey G, Lazarus JH, Dayan CM, Okosieme OE (2018). Global Epidemiology of Hyperthyroidism and Hypothyroidism. Nat Rev Endocrinol.

[33] Biondi B (2023). Subclinical Hypothyroidism in Patients with Obesity and Metabolic Syndrome: A Narrative Review. Nutrients.

[34] Grandone A, Santoro N, Coppola F, Calabrò P, Perrone L, Del Giudice EM (2010). Thyroid Function Derangement and Childhood Obesity: An Italian Experience. BMC Endocr Disord.

[35] Attilakos A, Garoufi A, Voudris K, Mastroyianni S, Fotinou A, Papadimitriou DT, Gavalakis N, Prassouli A, Katsarou E (2007). Thyroid Dysfunction Associated with Increased Low-Density Lipoprotein Cholesterol in Epileptic Children Treated with Carbamazepine Monotherapy: A Causal Relationship?. J. Eur. Paediatr. Neurol. Soc.

[36] Vainionpää LK, Mikkonen K, Rättyä J, Knip M, Pakarinen AJ, Myllylä VV, Isojärvi JIT (2004). Thyroid Function in Girls with Epilepsy with Carbamazepine, Oxcarbazepine, or Valproate Monotherapy and after Withdrawal of Medication. Epilepsia.

[37] Rabast U, Hahn A, Reiners C, Ehl M (1981). Thyroid Hormone Changes in Obese Subjects during Fasting and a Very-Low-Calorie Diet. Int J Obes.

[38] Araujo RL, Andrade BM, Padrón AS, Gaidhu MP, Perry RLS, Carvalho DP, Ceddia RB (2010). High-Fat Diet Increases Thyrotropin and Oxygen Consumption without Altering Circulating 3,5,3’-Triiodothyronine (T3) and Thyroxine in Rats: The Role of Iodothyronine Deiodinases, Reverse T3 Production, and Whole-Body Fat Oxidation. Endocrinology.

[39] Zhang X, Chen W, Shao S, Xu G, Song Y, Xu C, Gao L, Hu C, Zhao J (2018). A High-Fat Diet Rich in Saturated and Mono-Unsaturated Fatty Acids Induces Disturbance of Thyroid Lipid Profile and Hypothyroxinemia in Male Rats. Mol Nutr Food Res.

[40] Shao S, Zhao Y, Song Y, Xu C, Yang J, Xuan S, Yan H, Yu C, Zhao M, Xu J (2014). Dietary High-Fat Lard Intake Induces Thyroid Dysfunction and Abnormal Morphology in Rats. Acta Pharmacol Sin.

[41] Barrea L, Caprio M, Camajani E, Verde L, Perrini S, Cignarelli A, Prodam F, Gambineri A, Isidori AM, Colao A (2024). Ketogenic Nutritional Therapy (KeNuT)-a Multi-Step Dietary Model with Meal Replacements for the Management of Obesity and Its Related Metabolic Disorders: A Consensus Statement from the Working Group of the Club of the Italian Society of Endocrinology (SIE). J Endocrinol Invest.

[42] Ruiz-Pozo VA, Tamayo-Trujillo R, Cadena-Ullauri S, Frias-Toral E, Guevara-Ramírez P, Paz-Cruz E, Chapela S, Montalván M, Morales-López T, Simancas-Racines D (2023). The Molecular Mechanisms of the Relationship between Insulin Resistance and Parkinson’s Disease Pathogenesis. Nutrients.

[43] Zambrano AK, Cadena-Ullauri S, Guevara-Ramírez P, Frias-Toral E, Ruiz-Pozo VA, Paz-Cruz E, Tamayo-Trujillo R, Chapela S, Montalván M, Sarno G (2023). The Impact of a Very-Low-Calorie Ketogenic Diet in the Gut Microbiota Composition in Obesity. Nutrients.

[44] Mayorga-Ramos A, Barba-Ostria C, Simancas-Racines D, Guamán LP (2022). Protective Role of Butyrate in Obesity and Diabetes: New Insights. Front Nutr.

[45] Camajani E, Feraco A, Verde L, Moriconi E, Marchetti M, Colao A, Caprio M, Muscogiuri G, Barrea L (2023). Ketogenic Diet as a Possible Non-Pharmacological Therapy in Main Endocrine Diseases of the Female Reproductive System: A Practical Guide for Nutritionists. Curr Obes Rep.

[46] Barrea L, Caprio M, Tuccinardi D, Moriconi E, Di Renzo L, Muscogiuri G, Colao A, Savastano S (2022). Could Ketogenic Diet “Starve” Cancer? Emerging Evidence. Crit Rev Food Sci Nutr.

[47] O’Neill B, Raggi P (2019). The Ketogenic Diet: Pros and Cons. Atherosclerosis.

[48] Mentella MC, Scaldaferri F, Ricci C, Gasbarrini A, Miggiano GAD (2019). Cancer and Mediterranean Diet: A Review. Nutrients.

[49] Verde L, Barrea L, Docimo A, Savastano S, Colao A, Muscogiuri G (2023). Chronotype as a Predictor of Weight Loss and Body Composition Improvements in Women with Overweight or Obesity Undergoing a Very Low-Calorie Ketogenic Diet (VLCKD). Clin Nutr.

[50] Guevara-Ramírez P, Paz-Cruz E, Cadena-Ullauri S, Ruiz-Pozo VA, Tamayo-Trujillo R, Felix ML, Simancas-Racines D, Zambrano AK (2023). Molecular Pathways and Nutrigenomic Review of Insulin Resistance Development in Gestational Diabetes Mellitus. Front Nutr.

[51] Chapela SP, Simancas-Racines D, Montalvan M, Frias-Toral E, Simancas-Racines A, Muscogiuri G, Barrea L, Sarno G, Martínez PI, Reberendo MJ (2023). Signals for Muscular Protein Turnover and Insulin Resistance in Critically Ill Patients: A Narrative Review. Nutrients.

[52] Chérrez-Ojeda I, Simancas-Racines D, Greiding L, Tinoco-Morán I (2018). Metabolic syndrome and chronic urticaria. Rev. Alerg. Mex.

[53] Guevara-Ramírez P, Cadena-Ullauri S, Ruiz-Pozo VA, Tamayo-Trujillo R, Paz-Cruz E, Simancas-Racines D, Zambrano AK (2022). Genetics, Genomics, and Diet Interactions in Obesity in the Latin American Environment. Front Nutr.

[54] Sanyal D, Raychaudhuri M (2016). Hypothyroidism and Obesity: An Intriguing Link. Indian J Endocrinol Metab.

[55] Rosenbaum M, Hirsch J, Murphy E, Leibel RL (2000). Effects of Changes in Body Weight on Carbohydrate Metabolism, Catecholamine Excretion, and Thyroid Function. Am J Clin Nutr.

[56] Danforth E, Horton ES, O’Connell M, Sims EA, Burger AG, Ingbar SH, Braverman L, Vagenakis AG (1979). Dietary-Induced Alterations in Thyroid Hormone Metabolism during Overnutrition. J Clin Invest.

[57] Knudsen N, Laurberg P, Rasmussen LB, Bülow I, Perrild H, Ovesen L, Jørgensen T (2005). Small Differences in Thyroid Function May Be Important for Body Mass Index and the Occurrence of Obesity in the Population. J Clin Endocrinol Metab.

[58] Reinehr T, De Sousa G, Andler W (2006). Hyperthyrotropinemia in Obese Children Is Reversible after Weight Loss and Is Not Related to Lipids. J Clin Endocrinol Metab.

[59] Biondi B (2010). Thyroid and Obesity: An Intriguing Relationship. J Clin Endocrinol Metab.

[60] Tagliaferri M, Berselli ME, Calò G, Minocci A, Savia G, Petroni ML, Viberti GC, Liuzzi A (2001). Subclinical Hypothyroidism in Obese Patients: Relation to Resting Energy Expenditure, Serum Leptin, Body Composition, and Lipid Profile. Obes Res.

[61] Chomard P, Vernhes G, Autissier N, Debry G (1985). Serum Concentrations of Total T4, T3, Reverse T3 and Free T4, T3 in Moderately Obese Patients. Hum Nutr Clin Nutr.

[62] Nannipieri M, Cecchetti F, Anselmino M, Camastra S, Niccolini P, Lamacchia M, Rossi M, Iervasi G, Ferrannini E (2009). Expression of Thyrotropin and Thyroid Hormone Receptors in Adipose Tissue of Patients with Morbid Obesity and/or Type 2 Diabetes: Effects of Weight Loss. Int J Obes.

[63] Juiz-Valiña P, Cordido M, Outeiriño-Blanco E, Pértega S, Varela-Rodríguez BM, García-Brao MJ, Mena E, Pena-Bello L, Sangiao-Alvarellos S, Cordido F (2020). Central Resistance to Thyroid Hormones in Morbidly Obese Subjects Is Reversed after Bariatric Surgery-Induced Weight Loss. J Clin Med.

[64] Longhi S, Radetti G (2013). Thyroid Function and Obesity. JCRPE J Clin Res Pediatr Endocrinol.

[65] Wadden TA, Mason G, Foster GD, Stunkard AJ, Prange AJ (1990). Effects of a Very Low Calorie Diet on Weight, Thyroid Hormones and Mood. Int J Obes.

[66] Leung AM, Braverman LE (2014). Consequences of Excess Iodine. Nat Rev Endocrinol.

[67] Radetti G, Longhi S, Baiocchi M, Cassar W, Buzi F (2012). Changes in Lifestyle Improve Body Composition, Thyroid Function, and Structure in Obese Children. J Endocrinol Invest.

[68] Ladenson PW, Kristensen JD, Ridgway EC, Olsson AG, Carlsson B, Klein I, Baxter JD, Angelin B (2010). Use of the Thyroid Hormone Analogue Eprotirome in Statin-Treated Dyslipidemia. Obstet Gynecol Surv.

[69] Wang X, Gao X, Han Y, Zhang F, Lin Z, Wang H, Teng W, Shan Z (2021). Causal Association Between Serum Thyrotropin and Obesity: A Bidirectional, Mendelian Randomization Study. J Clin Endocrinol Metab.

[70] Ding X, Zhao Y, Zhu C-Y, Wu L-P, Wang Y, Peng Z-Y, Deji C, Zhao F-Y, Shi B-Y (2021). The Association between Subclinical Hypothyroidism and Metabolic Syndrome: An Update Meta-Analysis of Observational Studies. Endocr J.

[71] Alwan H, Ribero VA, Efthimiou O, Del Giovane C, Rodondi N, Duntas L (2023). A Systematic Review and Meta-Analysis Investigating the Relationship between Metabolic Syndrome and the Incidence of Thyroid Diseases. Endocrine.

[72] Wang B, Song R, He W, Yao Q, Li Q, Jia X, Zhang JA (2018). Sex Differences in the Associations of Obesity With Hypothyroidism and Thyroid Autoimmunity Among Chinese Adults. Front Physiol.

[73] van Hulsteijn LT, Pasquali R, Casanueva F, Haluzik M, Ledoux S, Monteiro MP, Salvador J, Santini F, Toplak H, Dekkers OM (2020). Prevalence of Endocrine Disorders in Obese Patients: Systematic Review and Meta-Analysis. Eur J Endocrinol.

[74] Marzullo P, Minocci A, Tagliaferri MA, Guzzaloni G, Di Blasio A, De Medici C, Aimaretti G, Liuzzi A (2010). Investigations of Thyroid Hormones and Antibodies in Obesity: Leptin Levels Are Associated with Thyroid Autoimmunity Independent of Bioanthropometric, Hormonal, and Weight-Related Determinants. J Clin Endocrinol Metab.

[75] Song R-H, Wang B, Yao Q-M, Li Q, Jia X, Zhang JA (2019). The Impact of Obesity on Thyroid Autoimmunity and Dysfunction: A Systematic Review and Meta-Analysis. Front Immunol.

[76] Biondi B, Kahaly GJ, Robertson RP (2019). Thyroid Dysfunction and Diabetes Mellitus: Two Closely Associated Disorders. Endocr Rev.

[77] Pasquali R, Casanueva F, Haluzik M, van Hulsteijn L, Ledoux S, Monteiro MP, Salvador J, Santini F, Toplak H, Dekkers OM (2020). European Society of Endocrinology Clinical Practice Guideline: Endocrine Work-up in Obesity. Eur J Endocrinol.

[78] Kaptein EM, Beale E, Chan LS (2009). Thyroid Hormone Therapy for Obesity and Nonthyroidal Illnesses: A Systematic Review. J Clin Endocrinol Metab.

[79] Mele C, Mai S, Cena T, Pagano L, Scacchi M, Biondi B, Aimaretti G, Marzullo P (2022). The Pattern of TSH and FT4 Levels across different BMI ranges in a large cohort of euthyroid patients with obesity. Obesity. Front. Endocrinol. (Lausanne)..

[80] Papoian V, Ylli D, Felger EA, Wartofsky L, Rosen JE (2019). Evaluation of Thyroid Hormone Replacement Dosing in Overweight and Obese Patients After a Thyroidectomy. Thyroid.

[81] Biondi B, Wartofsky L (2014). Treatment with Thyroid Hormone. Endocr Rev.

[82] Okosieme O, Gilbert J, Abraham P, Boelaert K, Dayan C, Gurnell M, Leese G, McCabe C, Perros P, Smith V (2016). Management of Primary Hypothyroidism: Statement by the British Thyroid Association Executive Committee. Clin Endocrinol (Oxf).

[83] Fallahi P, Ferrari SM, Camastra S, Politti U, Ruffilli I, Vita R, Navarra G, Benvenga S, Antonelli A (2017). TSH Normalization in Bariatric Surgery Patients After the Switch from L-Thyroxine in Tablet to an Oral Liquid Formulation. Obes Surg.

[84] Trimboli P, Ossola N, Torre A, Mongelli F, Quarenghi M, Camponovo C, Lucchini B, Rotondi M, Ruinelli L, Garofalo F (2023). The Performance of Levothyroxine Tablet Is Impaired by Bariatric Surgery. Endocrine.

[85] Eom YS, Wilson JR, Bernet VJ (2022). Links between Thyroid Disorders and Glucose Homeostasis. Diabetes Metab J.

[86] Pucci E, Chiovato L, Pinchera A (2000). Thyroid and Lipid Metabolism. J. Int. Assoc. Study Obes..

[87] Bellastella G, Scappaticcio L, Caiazzo F, Tomasuolo M, Carotenuto R, Caputo M, Arena S, Caruso P, Maiorino MI, Esposito K (2022). Mediterranean Diet and Thyroid: An Interesting Alliance. Nutrients.

[88] Leko MB, Gunjača I, Pleić N, Zemunik T (2021). Environmental Factors Affecting Thyroid-Stimulating Hormone and Thyroid Hormone Levels. Int. J. Mol. Sci..

[89] •• Brdar D, Gunjača I, Pleić N, Torlak V, Knežević P, Punda A, Polašek O, Hayward C, Zemunik T. The effect of food groups and nutrients on thyroid hormone levels in healthy individuals. Nutrition. 2021;91–2. 10.1016/j.nut.2021.111394. **This study explores the complex connection between nutrients and thyroid hormone levels. Using a meticulous approach, it relies on a comprehensive dietary survey involving 58 carefully chosen items and a substantial sample size of over 4,500 individuals. The findings provide valuable and statistically robust insights into the intricate interplay between nutrition and thyroid health.** 10.1016/j.nut.2021.111394 34303955

[90] Mathieson RA, Walberg JL, Gwazdauskas FC, Hinkle DE, Gregg JM (1986). The Effect of Varying Carbohydrate Content of a Very-Low-Caloric Diet on Resting Metabolic Rate and Thyroid Hormones. Metabolism.

[91] Kopp W (2004). Nutrition, Evolution and Thyroid Hormone Levels – a Link to Iodine Deficiency Disorders?. Med Hypotheses.

[92] Rebollo-Hernanz M, Zhang Q, Aguilera Y, Martín-Cabrejas MA, Gonzalez de Mejia E (2009). Relationship of the Phytochemicals from Coffee and Cocoa By-Products with Their Potential to Modulate Biomarkers of Metabolic Syndrome In Vitro.. Antioxidants (Basel, Switzerland).

[93] Pałkowska-Goździk E, Lachowicz K, Rosołowska-Huszcz D (2017). Effects of Dietary Protein on Thyroid Axis Activity. Nutrients.

[94] Larsen D, Singh S, Brito M (2022). Thyroid, Diet, and Alternative Approaches. J Clin Endocrinol Metab.

[95] Kose E, Guzel O, Demir K, Arslan N (2017). Changes of Thyroid Hormonal Status in Patients Receiving Ketogenic Diet Due to Intractable Epilepsy. J Pediatr Endocrinol Metab.

[96] Boelen A, Wiersinga WM, Fliers E (2008). Fasting-Induced Changes in the Hypothalamus-Pituitary-Thyroid Axis. Thyroid.

[97] Sirikonda NS, Patten WD, Phillips JR, Mullett CJ (2012). Ketogenic Diet: Rapid Onset of Selenium Deficiency-Induced Cardiac Decompensation. Pediatr Cardiol.

[98] Moriyama K, Watanabe M, Yamada Y, Shiihara T (2015). Protein-Losing Enteropathy as a Rare Complication of the Ketogenic Diet. Pediatr Neurol.

[99] Lee YJ, Nam SO, Kim KM, Kim YM, Yeon GM (2007). ongitudinal Change in Thyroid Hormone Levels in Children with Epilepsy on a Ketogenic Diet: Prevalence and Risk Factors.. J. epilepsy Res.

[100] Pasquali R, Baraldi G, Biso P, Pasqui F, Mattioli L, Capelli M, Callivá R, Spoto M, Melchionda N, Labò G (1983). Relationships between Iodothyronine Peripheral Metabolism and Ketone Bodies during Hypocaloric Dietary Manipulations. J Endocrinol Investig.

[101] Helwig NE, Hong S, Hsiao-wecksler ET (1993). Effect of Small Doses of Lodine on Thyroid Function During Caloric Restriction in Normal Subjects.. Deparrment Clin Endocrinol Med Clin Univ Essen, FRG.

[102] Serog P, Apfelbaum M, Autissier N, Baigts F, Brigant L, Ktorza A (1982). Effects of Slimming and Composition of Diets on VO2 and Thyroid Hormones in Healthy Subjects. Am J Clin Nutr.

[103] Volek JS, Sharman MJ, Love DM, Avery NG, Gómez AL, Scheett TP, Kraemer WJ (2002). Body Composition and Hormonal Responses to a Carbohydrate-Restricted Diet. Metabolism.

[104] Ünsal Y, Nalbanto Ö, Edizer S, Ak Z, Pekuz S. The effect of ketogenic diet on thyroid functions in children with drug-resistant epilepsy. 2021. 10.1007/s10072-021-05225-y 33846882

[105] Iacovides S, Meiring RM (2018). The Effect of a Ketogenic Diet versus a High-Carbohydrate, Low-Fat Diet on Sleep, Cognition, Thyroid Function, and Cardiovascular Health Independent of Weight Loss: Study Protocol for a Randomized Controlled Trial. Trials.

[106] Yılmaz Ü, Edizer S, Köse M, Akışin Z, Güzin Y, Pekuz S, Kırkgöz HH, Yavuz M, Ünalp A (2021). The Effect of Ketogenic Diet on Serum Lipid Concentrations in Children with Medication Resistant Epilepsy. Seizure.

[107] Cornu MG, Martinuzzi ALN, Roel P, Sanhueza L, Sepúlveda ME, Orozco MS, Sánchez CA, Gulino M (2020). Incidence of low-triiodothyronine syndrome in patients with septic shock. Rev Bras Ter intensiva.

[108] Hamed SA (2015). The Effect of Antiepileptic Drugs on Thyroid Hormonal Function: Causes and Implications. Expert Rev Clin Pharmacol.

[109] Verde L, Dalamaga M, Capó X, Annunziata G, Hassapidou M, Docimo A, Savastano S, Colao A, Muscogiuri G, Barrea L (2022). The Antioxidant Potential of the Mediterranean Diet as a Predictor of Weight Loss after a Very Low-Calorie Ketogenic Diet (VLCKD) in Women with Overweight and Obesity.. Antioxidants (Basel, Switzerland).

[110] Yılmaz Ü, Nalbantoğlu Ö, Güzin Y, Edizer S, Akışin Z, Pekuz S, Kırkgöz HH, Yavuz M, Ünalp A, Özkan B (2021). The Effect of Ketogenic Diet on Thyroid Functions in Children with Drug-Resistant Epilepsy. Neurol Sci.

[111] Kuroki R, Sadamoto Y, Imamura M, Abe Y, Higuchi K, Kato K, Koga T, Furue M (1999). Acanthosis Nigricans with Severe Obesity, Insulin Resistance and Hypothyroidism: Improvement by Diet Control. Dermatology.

[112] Khodabakhshi A, Seyfried TN, Kalamian M, Beheshti M, Davoodi SH (2020). Does a Ketogenic Diet Have Beneficial Effects on Quality of Life, Physical Activity or Biomarkers in Patients with Breast Cancer: A Randomized Controlled Clinical Trial. Nutr J.

[113] Gomez-Arbelaez D, Crujeiras AB, Castro AI, Martinez-Olmos MA, Canton A, Ordoñez-Mayan L, Sajoux I, Galban C, Bellido D, Casanueva FF (2018). Resting Metabolic Rate of Obese Patients under Very Low Calorie Ketogenic Diet. Nutr Metab.

